# Ventilatory efficiency during high-frequency oscillatory ventilation with volume guarantee in preterm infants

**DOI:** 10.1007/s00431-026-07188-8

**Published:** 2026-06-26

**Authors:** Kamal Ali, Mesaed Alsenani, Saad Alshareedah, Faisal Alamer, Piero Alberti, Abdulaziz Homedi, Saif Alsaif, Ibrahim Ali, Theodore Dassios, Anne Greenough

**Affiliations:** 1 Neonatal Intensive Care Department, King Abdulaziz Medical City, Riyadh, Saudi Arabia; 2 Neonatal Intensive Care Centre, King’s College Hospital, 4th Floor, Golden Jubilee Wing, Denmark Hill, London, SE5 9RS UK; 3 King Abdullah International Medical Research Centre, Riyadh, Saudi Arabia; 4 Department of Women and Children’s Health, School of Life Course Sciences, Faculty of Life Sciences and Medicine, King’s College London, London, UK; 5 College of Medicine, King Saud Bin Abdulaziz University for Health Sciences, Riyadh, Saudi Arabia

**Keywords:** Oscillatory ventilation, Ventilatory efficiency, Preterm infants

## Abstract

This study aims to evaluate the relationship between ventilator-derived carbon dioxide clearance and arterial CO_2_ elimination during high-frequency oscillatory ventilation with volume guarantee (HFOV-VG), and to examine determinants and clinical relevance of ventilatory efficiency. In this cohort study, preterm infants less than 30 weeks of gestation receiving HFOV-VG were studied during the first 72 h after birth. Minute-level ventilator data were paired with arterial blood gases. Ventilatory efficiency was defined as weight-normalised DCO_2_ divided by arterial PCO_2_. Temporal patterns and determinants were analysed using generalised estimating equations. The clinical outcome was death or moderate-to-severe bronchopulmonary dysplasia (BPD). A total of 185,018 ventilator observations and 595 paired ventilator–blood gas measurements from 60 infants were analysed. Arterial PCO_2_ remained stable (50 [43–56] mmHg) despite wide variation in DCO_2_ (34.6 [27.1–47.8]). Ventilatory efficiency varied between infants (0.72 [0.55–0.98]) and demonstrated a temporal pattern, decreasing from 0.77 (0.61–0.90) at 0–6 h to 0.65 (0.45–0.80) at 6–12 h and increasing to 0.76 (0.56–1.07) at 24–48 h. Higher efficiency was associated with higher oscillatory frequency (*p* < 0.001). Death or moderate-to-severe BPD occurred in 85.0%, 70.0%, and 45.0% across low, intermediate-, and high-efficiency tertiles, respectively. Ventilatory efficiency demonstrated moderate discrimination for the composite outcome (AUC 0.69), improving to 0.76 with FiO_2_ and mean airway pressure. *Conclusion*: During HFOV-VG, ventilator-derived CO_2_ clearance showed limited correspondence with arterial PCO_2_. Ventilatory efficiency had moderate discrimination for adverse respiratory outcomes.

**Table:** 

**What is Known:** •* During high-frequency oscillatory ventilation, ventilator-derived indices such as oscillatory tidal volume and DCO2 are commonly used to guide carbon dioxide clearance.* •* The relationship between ventilator-derived carbon dioxide transport and effective arterial CO2 elimination during HFOV with volume guarantee remains incompletely understood.*	
**What is New:** •* Ventilatory efficiency provides a physiological measure integrating ventilator-derived carbon dioxide transport and arterial CO2 elimination during HFOV with volume guarantee, a relationship that is not routinely evaluated in clinical practice.* •* Ventilatory efficiency demonstrated modest discrimination for death or moderate-to-severe bronchopulmonary dysplasia, with discrimination improving when combined with markers of respiratory support intensity.*

**What is Known:**

•* During high-frequency oscillatory ventilation, ventilator-derived indices such as oscillatory tidal volume and DCO2 are commonly used to guide carbon dioxide clearance.*

•* The relationship between ventilator-derived carbon dioxide transport and effective arterial CO2 elimination during HFOV with volume guarantee remains incompletely understood.*

**What is New:**

•* Ventilatory efficiency provides a physiological measure integrating ventilator-derived carbon dioxide transport and arterial CO2 elimination during HFOV with volume guarantee, a relationship that is not routinely evaluated in clinical practice.*

•* Ventilatory efficiency demonstrated modest discrimination for death or moderate-to-severe bronchopulmonary dysplasia, with discrimination improving when combined with markers of respiratory support intensity.*

## Introduction

High-frequency oscillatory ventilation (HFOV) is widely used in preterm infants to support gas exchange with the hope of limiting lung injury [1–3]. In clinical practice, ventilator-derived indices, particularly oscillatory tidal volume (VThf) and the diffusion coefficient of carbon dioxide (DCO_2_), are used to guide carbon dioxide clearance [4]. However, these measures reflect delivered oscillatory transport rather than effective alveolar ventilation, and their relationship to arterial carbon dioxide tension (PCO_2_) remains uncertain [5, 6].

Clinical observations suggest that similar levels of ventilator-derived CO_2_ clearance may be associated with different arterial CO_2_ levels [7]. This apparent uncoupling likely reflects interactions between delivered oscillatory ventilation and underlying lung physiology, including recruitment, ventilation–perfusion matching, airway resistance, and dead space [1, 8, 9]. Consequently, ventilator-derived indices may not reliably capture the efficiency with which delivered ventilation translates into effective gas exchange.

The introduction of volume guarantee (VG) during HFOV improves control of delivered tidal volume and reduces variability in oscillatory ventilation [10]. Nevertheless, variability in arterial CO_2_ persists despite similar ventilator settings, indicating that determinants of effective CO_2_ elimination extend beyond tidal volume alone [7]. This highlights a gap between ventilator-derived measures of CO_2_ transport and physiological gas exchange. To address this, we conceptualised ventilatory efficiency as the relationship between ventilator-derived CO_2_ clearance and arterial CO_2_ elimination.

In this study, we evaluated the relationship between ventilator-derived carbon dioxide transport and effective arterial CO_2_ elimination during high-frequency oscillatory ventilation with volume guarantee in preterm infants. Building on prior experimental work examining DCO_2_ and PaCO_2_ relationships during HFOV-VG [7], we defined ventilatory efficiency as the ratio of weight-normalised DCO_2_ to arterial PCO_2_ and examined its temporal behaviour, between-infant variability and association with clinically relevant respiratory outcomes in a clinical neonatal cohort. We additionally explored physiological and ventilatory factors associated with ventilatory efficiency using time-matched ventilator and arterial blood gas data.

## Methods

### Study design and setting

This retrospective observational physiological cohort study was conducted in a tertiary level III neonatal intensive care unit at King Abdulaziz Medical City, Riyadh, Saudi Arabia, between October 2024 and February 2026. Ethical approval was obtained from the King Abdullah International Medical Research Centre, which granted a waiver of informed consent due to the observational design and use of routinely collected clinical data. No additional interventions were performed for study purposes.

### Study population

Preterm infants born at less than 30 weeks of gestation who required invasive ventilation with high-frequency oscillatory ventilation with volume guarantee (HFOV-VG) during the early postnatal period were eligible. Infants were included if continuous ventilator data were available from HFOV-VG initiation and at least one arterial blood gas measurement was recorded within the first 72 h of life. Infants without paired ventilator–blood gas observations or with major congenital anomalies affecting cardiopulmonary physiology were excluded.

### Ventilatory management and data acquisition

All infants were ventilated using the Dräger VN600 ventilator (Dräger Medical, Lübeck, Germany) in HFOV-VG mode during the early postnatal period. Initial settings followed unit practice with a target oscillatory tidal volume (VThf) typically between 1.5 and 2.0 mL/kg and an oscillatory frequency between 15 and 18 Hz, adjusted according to gas exchange and clinical status. Mean airway pressure (MAP) was initiated at 10 mbar and titrated according to oxygenation.

Ventilator data were recorded directly from the ventilator at 1-min intervals and exported as continuous time-series data. Recorded variables included measured and set VThf, oscillatory amplitude, diffusion coefficient of carbon dioxide (DCO_2_), fraction of inspired oxygen (FiO_2_), oscillatory frequency, and MAP. Measured VThf was obtained directly from the ventilator and exported as part of the continuous ventilator dataset.

### Definition of ventilatory efficiency

Measured VThf and DCO_2_ were normalised to body weight (kg). Ventilatory efficiency was defined as weight-normalised DCO_2_ divided by the corresponding arterial PCO_2_ and was calculated for each paired ventilator–blood gas observation. Higher values therefore reflected greater ventilator-derived CO_2_ transport relative to arterial CO_2_ levels, indicating more efficient gas exchange.

### Data processing and paired observations

Ventilator data were processed separately for each infant. Time-stamped ventilator recordings were matched to arterial blood gas measurements obtained during routine clinical care within a predefined time window (± 10 min). This window was selected to maintain close temporal proximity between ventilator measurements and blood gas sampling while ensuring an adequate number of paired observations for analysis. When multiple ventilator values were available within this window, the closest value to the blood gas time was used. A paired observation was defined only when a PCO_2_ value was available within this time window. No interpolation was performed.

All analyses were restricted to the first 72 h following initiation of HFOV. Temporal patterns were examined across predefined time epochs (0–6, 6–12, 12–24, 24–48, and 48–72 h).

### Ventilatory efficiency phenotypes

Ventilatory efficiency was calculated for each paired ventilator–blood gas observation. For infant-level analyses, a single summary value was derived for each infant by calculating the median ventilatory efficiency across all paired observations within the first 72 h.

Infants were then categorised into low-efficiency, intermediate-efficiency, and high-efficiency groups based on tertiles of the distribution of their median VE values in our cohort. This categorisation was used to describe between-infant variability in ventilatory efficiency. Ventilatory efficiency was also analysed as a continuous variable in regression models.

### Measurements and outcomes

The primary physiological measurement was ventilatory efficiency during the first 72 h of HFOV-VG.

The main clinical outcome was the composite of death before 36 weeks of postmenstrual age or moderate-to-severe bronchopulmonary dysplasia at 36 weeks of postmenstrual age. Additional clinical outcomes included mortality, moderate-to-severe bronchopulmonary dysplasia, major intraventricular haemorrhage.

### Data management and statistical analysis

Data processing was performed using Python (Python Software Foundation). Statistical analyses were conducted using IBM SPSS Statistics version 31 (IBM Corp., Armonk, NY, USA).

Continuous variables are presented as median (interquartile range) and categorical variables as n/N (%). For infant-level analyses, ventilatory efficiency was summarised for each infant as the median value across all paired ventilator–blood gas observations within the first 72 h. Infants were categorised into low-efficiency, intermediate-efficiency and high-efficiency groups based on tertiles of these median values.

Comparisons across efficiency groups were performed using the Kruskal–Wallis test for continuous variables and the χ^2^ test or Fisher’s exact test for categorical variables, as appropriate.

To evaluate factors associated with ventilatory efficiency at the observation level while accounting for repeated measurements within infants, generalised estimating equation models were used with clustering at the infant level. Ventilatory efficiency was log-transformed for regression analyses to address skewness. Because ventilatory efficiency was log-transformed before modelling, regression coefficients were exponentiated and are presented as adjusted ratios, representing proportional changes in ventilatory efficiency associated with each predictor.

The multivariate model included measured VThf/kg, weight-normalised DCO_2_, oscillatory frequency, FiO_2_, time from HFOV initiation and gestational age. Results are presented as adjusted ratios with 95% confidence intervals. Covariates were selected a priori based on physiological relevance and previously reported associations with gas exchange during HFOV rather than by univariable screening. Variables represented delivered oscillatory ventilation (VThf/kg and weight-normalised DCO_2_), ventilator strategy (oscillatory frequency), respiratory disease severity (FiO_2_), temporal adaptation following HFOV initiation (time from HFOV initiation), and developmental maturity (gestational age). Multicollinearity was assessed using variance inflation factors, and all values were below 5, indicating no evidence of problematic collinearity.

Associations between infant-level ventilatory efficiency and clinical outcomes were examined using logistic regression. The primary clinical outcome was the composite of death before 36 weeks of postmenstrual age or moderate-to-severe bronchopulmonary dysplasia at 36 weeks of postmenstrual age. Ventilatory efficiency was analysed both as a continuous variable, per 0.1-unit increase, and by tertiles, using the high-efficiency group as the reference. Models were adjusted for first 24-h FiO_2_, antenatal steroid exposure and sex, with an additional sensitivity model including gestational age.

Discriminatory performance of ventilatory efficiency for the composite outcome was assessed using receiver operating characteristic curve analysis. ROC curves were generated for ventilatory efficiency as a continuous variable and for categorical models based on efficiency tertiles. Additional models incorporating ventilatory efficiency with early respiratory support variables, including first 24-h FiO_2_ and mean airway pressure, were also evaluated. Discrimination was quantified using the area under the ROC curve with 95% confidence intervals. Given the sample size and absence of external validation, these analyses were considered exploratory.

Analyses were performed using available-case data, and two-sided *p*-values < 0.05 were considered statistically significant.

## Results

Table 1 summarises the cohort characteristics and physiological measurements. The median gestational age was 26.0 (25.0–28.0) weeks and birth weight was 0.86 (0.70–1.00) kg. HFOV-VG was initiated at a median postnatal age of 2 (1–4) h, with a median FiO_2_ of 0.35 (0.30–0.55), MAP of 10.0 (9.0–11.0) mbar, and CRIB-II score of 9 (7–11). A total of 185,018 min-level ventilator observations and 595 paired ventilator–blood gas observations from 60 infants were available for analysis. At paired observations, the median measured VThf/kg was 1.70 (1.50–1.95) mL/kg, weight-normalised DCO_2_ was 34.6 (27.1–47.8), arterial PCO_2_ was 50 (43–56) mmHg, and ventilatory efficiency index was 0.72 (0.55–0.98).

**Table 1:** Cohort characteristics and signal burden during the first 72 h of HFOV with volume guarantee

Characteristic	Value
Study cohort	
Gestational age, weeks	26.0 (25.0–28.0)
Birth weight, kg	0.86 (0.70–1.00)
Postnatal age at HFOV-VG initiation, h	2 (1–4)
Caesarean delivery, *n* (%)	37/60 (61.7%)
5-min Apgar score	7 (6–8)
Primary diagnosis requiring HFOV-VG, *n* (%)	Respiratory distress syndrome, 60/60 (100%)
FiO_2_ at HFOV-VG initiation, %	0.35 (0.30–0.55)
MAP at HFOV-VG initiation, mbar	10.0 (9.0–11.0)
CRIB-II score	9 (7–11)
Data density	
Minute-level ventilator observations, *n*	185,018
Infants with paired ventilator–blood gas data, *n*	60
Paired ventilator–blood gas observations, *n*	595
Paired blood gases per infant, *n*	10 (8–12)
Variables at paired observations	
Measured VThf/kg, mL/kg	1.70 (1.50–1.95)
Weight-normalised DCO_2_	34.6 (27.1–47.8)
PCO_2_, mmHg	50 (43–56)
Ventilatory efficiency index	0.72 (0.55–0.98)

Values are presented as median (interquartile range) or *n*/*N* (%) unless stated otherwise

*MAP* mean airway pressure, *VThf* high-frequency tidal volume, *DCO*_*2*_ diffusion coefficient of carbon dioxide, *PCO*_*2*_ partial pressure of carbon dioxide, *CRIB-II* clinical risk index for babies II

Table 2 demonstrates temporal changes in ventilatory efficiency during the first 72 h of HFOV-VG. Ventilatory efficiency was highest during the first 6 h, decreased at 6–12 h, and subsequently increased between 12 and 48 h before stabilising. In contrast, arterial PCO_2_ remained relatively stable across epochs despite changes in DCO_2_ and measured VThf/kg. Oscillatory frequency was maintained within a narrow range (15–17 Hz) throughout the study period, suggesting that temporal changes in ventilatory efficiency were unlikely to be explained by variation in frequency. Mean airway pressure and FiO_2_ decreased progressively over time, reflecting improving respiratory status and reduced oxygenation requirements.

**Table 2:** Temporal evolution of ventilatory efficiency and component variables during the first 72 h of HFOV with volume guarantee

Epoch (h)	Paired observations (*n*)	VE index	PCO_2_ (mmHg)	DCO_2_/kg	Frequency (Hz)	Measured VThf/kg (mL/kg)	MAP (mbar)	FiO_2_ (%)
0–6	17	0.77 (0.61–0.90)	53 (43–64)	32.0 (29.6–50.5)	15–17	1.67 (1.50–1.85)	13 (11–14)	65 (30–90)
6–12	32	0.65 (0.45–0.80)	47 (42–60)	26.8 (23.5–32.9)	15–17	1.55 (1.47–1.80)	11 (10–12)	42 (32–70)
12–24	147	0.71 (0.55–0.99)	47 (40–54)	32.2 (26.1–46.3)	15–17	1.67 (1.47–1.91)	11 (10–12.5)	40 (28–60)
24–48	240	0.76 (0.56–1.07)	49 (43–55)	35.9 (27.4–51.7)	15–17	1.75 (1.50–2.00)	11 (10–12)	30 (25–37)
48–72	155	0.72 (0.53–0.96)	51 (48–56)	35.2 (27.8–46.3)	15–17	1.71 (1.50–2.00)	10 (9–11.5)	30 (25–40)

Values are median (interquartile range). Paired observations represent time-matched ventilator and arterial blood gas measurements

Table 3 summarises infant-level ventilatory efficiency phenotypes and clinical outcomes. Infants in the high-efficiency group had higher gestational age and birth weight than those in the low-efficiency group (*p* = 0.015 and *p* < 0.001, respectively). Mean airway pressure differed modestly between groups (*p* = 0.016). Moderate-to-severe BPD occurred in 85.0%, 60.0%, and 45.0% of the low-, intermediate-, and high-efficiency groups, respectively (*p* = 0.028). Similarly, the composite outcome of death before 36 weeks of PMA or moderate-to-severe BPD occurred more frequently in the low-efficiency group (85.0%) than in the intermediate (70.0%) and high-efficiency groups (45.0%; *p* = 0.047).

**Table 3:** Infant-level ventilatory efficiency phenotypes and respiratory outcomes

Characteristic	Low efficiency (*n* = 20)	Intermediate efficiency (*n* = 20)	High efficiency (*n* = 20)	*p*-value
Baseline characteristics				
Gestational age, weeks	26.0 (24.0–27.2)	26.0 (25.0–27.0)	27.0 (26.0–29.0)	0.015
Birth weight, g	765 (592–842)	830 (645–912)	1075 (972–1385)	< 0.001
Paired blood gases per infant, n	11 (8–13)	10 (8–12)	10 (8–12)	0.776
Physiological characteristics				
Median ventilatory efficiency index	0.56 (0.48–0.59)	0.72 (0.66–0.78)	1.10 (0.93–1.38)	< 0.001
Measured VThf/kg, mL/kg	1.51 (1.37–1.66)	1.74 (1.62–1.88)	1.87 (1.70–2.04)	< 0.001
Weight-normalised DCO_2_	26.3 (20.0–28.4)	34.9 (32.7–40.6)	53.4 (49.8–77.8)	< 0.001
Arterial PCO_2_, mmHg	48.8 (45.8–50.0)	50.0 (46.8–53.2)	52.8 (46.0–55.2)	0.276
Mean airway pressure, mbar	10.0 (9.0–11.1)	11.0 (10.0–12.0)	11.0 (11.0–13.6)	0.016
Clinical outcomes				
Death before 36 weeks of PMA	0/19 (0.0%)	6/20 (30.0%)	0/20 (0.0%)	0.001
Moderate-to-severe BPD at 36 weeks of PMA	17/20 (85.0%)	12/20 (60.0%)	9/20 (45.0%)	0.028
Death before 36 weeks of PMA or moderate-to-severe BPD	17/20 (85.0%)	14/20 (70.0%)	9/20 (45.0%)	0.047
Major intraventricular haemorrhage	6/20 (30.0%)	5/20 (25.0%)	4/20 (20.0%)	0.766
Retinopathy of prematurity	7/20 (35.0%)	5/20 (25.0%)	2/20 (10.0%)	0.170
Culture-positive sepsis	3/20 (15.0%)	3/20 (15.0%)	2/20 (10.0%)	0.866
Postnatal corticosteroid exposure	13/20 (65.0%)	8/20 (40.0%)	8/20 (40.0%)	0.189

Values are presented as median (interquartile range) or *n*/*N* (%)

Table 4 shows the multivariate model for ventilatory efficiency during the first 72 h of HFOV-VG. Higher oscillatory frequency and weight-normalised DCO_2_ were independently associated with higher ventilatory efficiency (both *p* < 0.001), whereas higher FiO_2_ and longer time from HFOV initiation were associated with lower efficiency (both *p* ≤ 0.001). Measured VThf/kg and gestational age were not independently associated with ventilatory efficiency.

**Table 4:** Multivariate model for row-level ventilatory efficiency during the first 72 h of HFOV-VG

Predictor	Adjusted ratio (95% CI)	*p*-value
Measured VThf/kg, per 0.5 mL/kg	1.11 (1.00–1.25)	0.061
Weight-normalised DCO_2_, per 5 units	1.09 (1.06–1.13)	< 0.001
Frequency, per 1 Hz	1.06 (1.03–1.09)	< 0.001
FiO_2_, per 10%	0.97 (0.96–0.98)	< 0.001
Time from HFOV start, per 10 h	0.97 (0.96–0.99)	0.001
Gestational age, per week	1.00 (0.96–1.05)	0.907

Adjusted ratios represent exponentiated coefficients from the generalised estimating equation model of log-transformed ventilatory efficiency. Values greater than 1 indicate a proportional increase in ventilatory efficiency, whereas values less than 1 indicate a proportional decrease. For example, an adjusted ratio of 1.06 for frequency indicates an approximately 6% higher ventilatory efficiency for each 1 Hz increase in oscillatory frequency

Table 5 shows the predictive performance of ventilatory efficiency for death before 36 weeks of postmenstrual age or moderate-to-severe bronchopulmonary dysplasia. Ventilatory efficiency alone demonstrated modest discrimination (AUC, 0.69; 95% CI, 0.55–0.82). Discrimination improved when ventilatory efficiency was combined with markers of respiratory support intensity, with the highest AUC observed for the model incorporating ventilatory efficiency, FiO_2_ and mean airway pressure (AUC, 0.76; 95% CI, 0.63–0.88).

**Table 5:** Predictive performance of ventilatory efficiency for adverse respiratory outcome

Model	AUC	95% CI	Sensitivity (%)	Specificity (%)	*p*-value
Ventilatory efficiency (continuous)	0.69	0.55–0.82	58	78	0.032
Low- vs high-efficiency tertile	0.66	0.54–0.78	52	80	0.041
Intermediate vs high tertile	0.61	0.49–0.73	48	76	0.118
Efficiency + FiO_2_ (first 24 h)	0.73	0.60–0.85	65	78	0.021
Efficiency + MAP (first 24 h)	0.71	0.58–0.83	61	75	0.028
Efficiency + FiO_2_ + MAP	0.76	0.63–0.88	70	76	0.015

Outcome was defined as death before 36 weeks’ postmenstrual age or moderate-to-severe bronchopulmonary dysplasia

*AUC* area under the receiver operating characteristic curve, *CI* confidence interval, *MAP* mean airway pressure

## Discussion

In this cohort of preterm infants less than 30 weeks of gestation ventilated on high-frequency oscillatory ventilation with volume guarantee, ventilator-derived indices of carbon dioxide clearance showed limited correspondence with arterial CO_2_ levels, indicating that ventilator-derived CO_2_ transport does not fully reflect the effectiveness of gas exchange. Lower ventilatory efficiency was associated with higher rates of moderate-to-severe bronchopulmonary dysplasia and the composite outcome of death before 36 weeks of postmenstrual age or moderate-to-severe bronchopulmonary dysplasia across efficiency strata. Ventilatory efficiency also demonstrated modest discrimination for adverse respiratory outcomes, which improved when combined with markers of respiratory support intensity. These findings suggest that ventilatory efficiency captures clinically relevant physiological information beyond ventilator-derived measures alone.

These findings are consistent with previous work by Belteki and Morley, who demonstrated using high-resolution ventilator datasets that weight-corrected VThf and DCO_2_ show only limited correspondence with arterial PCO_2_ during HFOV-VG, despite stable delivery of target tidal volume over time [11]. Their observations suggest that even when oscillatory tidal volume is tightly controlled, ventilator-derived measures of CO_2_ transport do not fully reflect effective gas exchange. The present study builds on these observations by demonstrating variability between infants in the relationship between DCO_2_ and arterial PCO_2_ under comparable ventilator conditions, indicating that the efficiency with which delivered ventilation translates into arterial CO_2_ elimination differs across individuals.

The dissociation between ventilator-derived CO_2_ transport and arterial CO_2_ elimination likely reflects the interaction between delivered oscillatory ventilation and heterogeneous lung physiology in preterm infants. Gas exchange during HFOV is mediated by multiple interacting mechanisms, including bulk convection, asymmetric velocity profiles, Taylor dispersion, and molecular diffusion [1, 5, 12], but the efficiency of these processes depends on the proportion of ventilation reaching effectively perfused alveoli [13, 14]. Ventilator-derived indices such as DCO_2_ incorporate oscillatory volume and frequency but do not account for regional heterogeneity in lung aeration or ventilation distribution [1, 13, 15, 16]. In partially recruited or heterogeneous lungs, a proportion of delivered oscillatory volume may contribute to dead space ventilation rather than effective alveolar gas exchange [19, 20]. Additional factors, including endotracheal tube resistance and circuit-related losses, may further reduce effective distal volume delivery, particularly in very preterm infants [2, 17]. Lung volume is central to this relationship, as HFOV is highly sensitive to lung recruitment; both underinflation and overdistension impair gas exchange efficiency [1, 18]. In inadequately recruited lungs, increases in oscillatory amplitude or DCO_2_ may not translate into improved CO_2_ elimination, whereas in more homogeneous and optimally recruited lungs, effective gas exchange may be achieved at lower levels of oscillatory transport. The association of ventilatory efficiency with FiO_2_ and time from HFOV initiation in the present study supports this interpretation, as both variables are indirect markers of lung recruitment and evolving respiratory disease.

Building on these physiological considerations, we evaluated ventilatory efficiency as a composite physiological index integrating ventilator-derived CO_2_ transport with arterial CO_2_ elimination. Ventilatory efficiency demonstrated variability across infants, indicating that effective CO_2_ elimination depends not only on delivered oscillatory volume but also on its distribution to perfused lung units. This interpretation is consistent with prior work showing that increases in oscillatory volume and frequency increase ventilator-derived CO_2_ transport without necessarily resulting in proportional reductions in arterial PCO_2_ [2, 9, 11, 19, 20]. Ventilatory efficiency also behaved as a relatively stable characteristic at the individual infant level over time, suggesting that it reflects underlying lung mechanics rather than short-term ventilator adjustments. Infants with lower efficiency had lower gestational age and birth weight, consistent with reduced lung compliance, less uniform lung aeration, and greater ventilation–perfusion mismatch in very preterm infants [21–24]. These findings support the interpretation that ventilatory efficiency represents an integrated physiological marker rather than a purely ventilator-derived variable.

The predictive analyses further evaluated the clinical relevance of ventilatory efficiency. Ventilatory efficiency demonstrated modest discrimination for the composite outcome of death before 36 weeks of postmenstrual age or moderate-to-severe bronchopulmonary dysplasia, with discrimination improving when combined with markers of respiratory support intensity, particularly FiO_2_ and mean airway pressure. These findings suggest that ventilatory efficiency may provide complementary physiological information alongside conventional measures of respiratory disease severity rather than functioning as an isolated predictor. The observed improvement in discrimination when ventilatory efficiency was interpreted within the broader context of respiratory support intensity further supports its role as an integrated physiological marker during HFOV-VG.

The use of frequencies above 15 Hz in our unit reflects an HFOV-VG strategy aimed at maintaining effective carbon dioxide elimination while minimising oscillatory tidal volume exposure. Previous physiological and clinical studies have demonstrated that higher oscillatory frequencies permit adequate carbon dioxide clearance with lower VThf, potentially reducing volutrauma in the immature lung. González-Pacheco and colleagues demonstrated the feasibility of using very high frequencies combined with low oscillatory tidal volumes during HFOV-VG while maintaining stable carbon dioxide elimination [9], and subsequent clinical studies reported adequate ventilation using frequencies of 15–18 Hz with VThf values around 1.5–2.0 mL/kg in extremely preterm infants [25, 26]. Our finding that higher oscillatory frequency was independently associated with greater ventilatory efficiency is consistent with this physiological concept, although prospective studies are required to determine the optimal frequency strategy during HFOV-VG.

From a clinical perspective, these findings indicate that ventilator-derived indices such as DCO_2_ should be interpreted in the context of the overall physiological response rather than as direct surrogates of effective CO_2_ elimination. Similar levels of oscillatory CO_2_ transport may be associated with different arterial PCO_2_ values across infants, reflecting variability in gas exchange efficiency. The temporal evolution of ventilatory efficiency observed in this study may similarly reflect dynamic changes in transitional lung physiology during the early postnatal period. The initial decrease in efficiency during the 6- to 12-h period followed by subsequent improvement may relate to evolving lung recruitment, surfactant effects, changing pulmonary fluid balance, and progressive stabilisation of respiratory mechanics over time. An efficiency-based approach may help clinicians recognise when increases in ventilator-derived CO_2_ transport do not translate into proportional improvements in arterial CO_2_ elimination, reflecting the influence of underlying lung physiology on effective gas exchange. In such situations, further escalation of ventilator settings may have limited benefit and should prompt reassessment of lung recruitment, including optimisation of mean airway pressure, and evaluation of factors affecting ventilation–perfusion matching. Its association with respiratory morbidity across efficiency strata suggests that ventilatory efficiency may serve as a complementary physiological marker to support interpretation of gas exchange efficiency during HFOV-VG rather than functioning solely as a ventilator-derived measure of CO_2_ transport.

The strengths of this study include the use of high-resolution, minute-level ventilator data and the direct pairing of ventilator parameters with concurrent arterial blood gases, allowing precise, time-matched physiological assessment of gas exchange. Consistent weight normalisation of key variables enabled comparison across infants of differing size and gestational maturity, and the large number of paired observations provided robust within-infant and between-infant analyses. Several limitations should be considered. Although the single-centre design may limit generalisability to units with different patient populations, ventilatory practices or ventilator platforms, the use of a consistent HFOV-VG strategy within a single tertiary neonatal centre reduced variability in ventilator management and data acquisition. The retrospective design limited the ability to account fully for clinician-driven ventilator adjustments; however, the use of high-resolution time-matched ventilator and arterial blood gas data enabled detailed physiological assessment across a large number of paired observations. Ventilatory efficiency, as defined in this study, represents a derived physiological index rather than a direct measure of alveolar ventilation or ventilation–perfusion matching, and further evaluation across different HFOV ventilator systems is required before broader clinical application. In addition, the outcome analyses were exploratory and based on a relatively modest sample size and therefore should be interpreted cautiously. Prospective studies incorporating continuous transcutaneous carbon dioxide monitoring alongside periodic arterial blood gas measurements may provide a more comprehensive assessment of carbon dioxide elimination and allow further validation of ventilatory efficiency during HFOV-VG. Further studies are required to determine whether ventilatory efficiency provides incremental physiological or clinical value beyond conventional measures of respiratory disease severity during HFOV-VG.

## Conclusions

In preterm infants ventilated with high-frequency oscillatory ventilation and volume guarantee, ventilator-derived indices of carbon dioxide clearance showed limited correspondence with arterial CO_2_ levels.

Ventilatory efficiency provided an integrated physiological measure of gas exchange and demonstrated modest discrimination for adverse respiratory outcomes, particularly when combined with markers of respiratory support intensity.

## Data Availability

The datasets generated and analyzed during the current study are available from the corresponding author upon reasonable request, subject to appropriate institutional approvals.

## References

[1] Pillow JJ (2005) High-frequency oscillatory ventilation: mechanisms of gas exchange and lung mechanics. Crit Care Med 33(3 Suppl):S135–S141 15753719 10.1097/01.ccm.0000155789.52984.b7

[2] Courtney SE, Durand DJ, Asselin JM, Hudak ML, Aschner JL, Shoemaker CT et al (2002) High-frequency oscillatory ventilation versus conventional mechanical ventilation for very-low-birth-weight infants. N Engl J Med 347(9):643–652 12200551 10.1056/NEJMoa012750

[3] Johnson AH, Peacock JL, Greenough A, Marlow N, Limb ES, Marston L et al (2002) High-frequency oscillatory ventilation for the prevention of chronic lung disease of prematurity. N Engl J Med 347(9):633–642 12200550 10.1056/NEJMoa020432

[4] Belteki G, Lin B, Morley CJ (2017) Weight-correction of carbon dioxide diffusion coefficient (DCO(2) ) reduces its inter-individual variability and improves its correlation with blood carbon dioxide levels in neonates receiving high-frequency oscillatory ventilation. Pediatr Pulmonol 52(10):1316–1322 28682001 10.1002/ppul.23759

[5] Fredberg JJ, Glass GM, Boynton BR, Frantz ID, 3rd. (1987) Factors influencing mechanical performance of neonatal high-frequency ventilators. J Appl Physiol (1985) 62(6):2485–90 10.1152/jappl.1987.62.6.2485 3475269

[6] Keszler M (2007) Strategy matters. Am J Perinatol 24(3):147–148 17372856 10.1055/s-2007-972929

[7] Gonzalez-Pacheco N, Sanchez-Luna M, Arribas-Sanchez C, Santos-Gonzalez M, Orden-Quinto C, Tendillo-Cortijo F (2020) DCO(2)/PaCO(2) correlation on high-frequency oscillatory ventilation combined with volume guarantee using increasing frequencies in an animal model. Eur J Pediatr 179(3):499–506 31823075 10.1007/s00431-019-03503-8

[8] Jobe AH, Bancalari E (2001) Bronchopulmonary dysplasia. Am J Respir Crit Care Med 163(7):1723–1729 11401896 10.1164/ajrccm.163.7.2011060

[9] González-Pacheco N, Sánchez-Luna M, Ramos-Navarro C, Navarro-Patiño N, de la Blanca AR (2016) Using very high frequencies with very low lung volumes during high-frequency oscillatory ventilation to protect the immature lung. A pilot study J Perinatol 36(4):306–310 26741575 10.1038/jp.2015.197

[10] Tuzun F, Deliloglu B, Cengiz MM, Iscan B, Duman N, Ozkan H (2020) Volume guarantee high-frequency oscillatory ventilation in preterm infants with RDS: tidal volume and DCO(2) levels for optimal ventilation using open-lung strategies. Front Pediatr 8:105 32266185 10.3389/fped.2020.00105 PMC7105735

[11] Belteki G, Morley CJ (2019) High-frequency oscillatory ventilation with volume guarantee: a single-centre experience. Arch Dis Child Fetal Neonatal Ed 104(4):F384–F389 30217870 10.1136/archdischild-2018-315490

[12] Chang HK (1984) Mechanisms of gas transport during ventilation by high-frequency oscillation. J Appl Physiol 56(3):553–563 6368498 10.1152/jappl.1984.56.3.553

[13] Froese AB, Bryan AC (1987) High frequency ventilation. Am Rev Respir Dis 135(6):1363–1374 3296894 10.1164/arrd.1987.135.6.1363

[14] Froese AB, Butler PO, Fletcher WA, Byford LJ (1987) High-frequency oscillatory ventilation in premature infants with respiratory failure: a preliminary report. Anesth Analg 66(9):814–824 3304021

[15] Froese AB (1987) High frequency ventilation. Can J Anaesth 34(Suppl 1):S37-40 20640737 10.1007/BF03009896

[16] Clark RH, Gerstmann DR, Null DM Jr, deLemos RA (1992) Prospective randomized comparison of high-frequency oscillatory and conventional ventilation in respiratory distress syndrome. Pediatrics 89(1):5–12 1728021

[17] Tingay DG, Mills JF, Morley CJ, Pellicano A, Dargaville PA (2013) Indicators of optimal lung volume during high-frequency oscillatory ventilation in infants. Crit Care Med 41(1):237–244 23269129 10.1097/CCM.0b013e31826a427a

[18] van Kaam AH, Rimensberger PC (2007) Lung-protective ventilation strategies in neonatology: what do we know–what do we need to know? Crit Care Med 35(3):925–931 17255875 10.1097/01.CCM.0000256724.70601.3A

[19] Pillow JJ, Sly PD, Hantos Z, Bates JH (2002) Dependence of intrapulmonary pressure amplitudes on respiratory mechanics during high-frequency oscillatory ventilation in preterm lambs. Pediatr Res 52(4):538–544 12357048 10.1203/00006450-200210000-00013

[20] Habib RH, Pyon KH, Courtney SE (2002) Optimal high-frequency oscillatory ventilation settings by nonlinear lung mechanics analysis. Am J Respir Crit Care Med 166(7):950–953 12359652 10.1164/rccm.200205-398OC

[21] Jobe AH (2011) The new bronchopulmonary dysplasia. Curr Opin Pediatr 23(2):167–172 21169836 10.1097/MOP.0b013e3283423e6b PMC3265791

[22] Pillow JJ, Musk GC, McLean CM, Polglase GR, Dalton RG, Jobe AH et al (2011) Variable ventilation improves ventilation and lung compliance in preterm lambs. Intensive Care Med 37(8):1352–1359 21567115 10.1007/s00134-011-2237-x

[23] Bancalari E, Jain D (2019) Bronchopulmonary dysplasia: 50 years after the original description. Neonatology 115(4):384–391 30974430 10.1159/000497422

[24] Thébaud B, Goss KN, Laughon M, Whitsett JA, Abman SH, Steinhorn RH et al (2019) Bronchopulmonary dysplasia. Nat Rev Dis Primers 5(1):78 31727986 10.1038/s41572-019-0127-7 PMC6986462

[25] Solís-García G, González-Pacheco N, Ramos-Navarro C, Rodríguez Sánchez de la Blanca A, Sánchez-Luna M (2021) Target volume-guarantee in high-frequency oscillatory ventilation for preterm respiratory distress syndrome: low volumes and high frequencies lead to adequate ventilation. Pediatr Pulmonol 56(8):2597–2603 34107176 10.1002/ppul.25529

[26] Sánchez-Luna M, González-Pacheco N, Belik J, Santos M, Tendillo F (2018) New ventilator strategies: high-frequency oscillatory ventilation combined with volume guarantee. Am J Perinatol 35(6):545–548 29694993 10.1055/s-0038-1637763

